# The association of visceral fat metabolism score with hyperuricemia—evidence from NHANES 1999–2018

**DOI:** 10.3389/fnut.2024.1497529

**Published:** 2025-01-10

**Authors:** Lin Xie, Huali Qu, Dandan Lai, Juan Li, Xushan Chen, Jiajia Xie

**Affiliations:** ^1^The Seventh Clinical Medical College of Guangzhou University of Chinese Medicine, Shenzhen, China; ^2^Shenzhen Bao’an Chinese Medicine Hospital, Guangzhou University of Chinese Medicine, Shenzhen, China

**Keywords:** hyperuricemia, visceral fat, cross-sectional survey, propensity score matching, NHANES

## Abstract

**Objectives:**

Despite substantial evidence that visceral obesity is an epidemiological risk factor for hyperuricemia (HUA), studies on the connection between the Metabolic Score for Visceral Fat (METS-VF) and HUA remain insufficient. This research focused on METS-VF’s potential role as a risk factor for HUA.

**Methods:**

Notably, 8,659 participants from the National Health and Nutrition Examination Survey (NHANES) from 1999 to 2018 were enrolled in this study. Propensity score matching (PSM), multivariate logistic regression analysis, subgroup analysis, interaction test, and restricted cubic spline (RCS) analysis were implemented to identify the correlation between METS-VF and HUA.

**Results:**

In the fully adjusted model, the results of the multiple logistic regression analysis indicated that METS-VF was related to an elevated prevalence of HUA [before PSM: odds ratio (OR) = 3.51 (2.88, 4.27), *p* < 0.001; after PSM: OR = 2.90 (2.36, 3.58), *p* < 0.001]. In RCS analysis, a non-linear positive correlation was observed between METS-VF and the incidence of HUA (before PSM: *p*-non-linear <0.001; after PSM: *p*-non-linear = 0.0065). Subgroup analysis and interaction tests revealed that the impact of METS-VF on HUA was modified by sex and ethnicity.

**Conclusion:**

There is a significant positive correlation between METS-VF and HUA in adults in the United States. METS-VF could serve as a valuable metric for assessing the development and progression of HUA.

## Introduction

Hyperuricemia (HUA) is a purine metabolism disorder distinguished by abnormally elevated serum uric acid levels (1). In the United States (US), approximately 47.1 million adults have HUA (2). In China, the prevalence of HUA is on the rise, with 14% of adults affected (3). HUA has become a significant global public health issue linked to gout and various other conditions (4–7).

Obesity, a worldwide pandemic, impacts over one-third of the global population (8). By 2025, it is anticipated that obesity will reach 18% in males and 21% in females (9). Compelling evidence indicates that obesity is a distinct and influential factor in the onset of HUA (10). Body mass index (BMI) is a standard physical measure to assess adiposity (11). However, BMI could not accurately assess fat and muscle mass (12). Waist circumference (WC) and waist-to-height ratio (WHtR) are utilized as indicators of central obesity to measure the distribution of body fat (13, 14). The limitation of WC is that it is not only affected by age and sex, but it also does not consider the individual’s height (15, 16). WHtR is not a suitable indicator for the surveillance of obesity in the elderly population in the prediction of central obesity (17). In addition, there is an interaction between visceral obesity and insulin resistance (IR) (18–20). Therefore, developing a comprehensive evaluation of visceral fat metabolism is crucial than using a single index to assess visceral fat accumulation. Metabolic Score for Visceral Fat (METS-VF) is an innovative measure of abdominal fat comprised of BMI, WHtR, Metabolic Score for Insulin Resistance (METS-IR), age, and sex (21). Previous studies have indicated that METS-VF serves as a superior predictor of HUA risk compared to the triglyceride-to-low-density lipoprotein cholesterol ratio, the triglyceride-to-glucose (TyG) index, the TyG-to-body mass index ratio, the TyG-to-waist-circumference ratio, and the insulin resistance metabolic score (22). In addition, research has demonstrated that METS-VF has links with chronic renal disease (23), erectile dysfunction (24), diabetes (25), and kidney stones (26). Currently, no research has investigated the link between METS-VF and HUA in the US population. We postulated a positive correlation between METS-VF and HUA.

## Methods

### Study population

**Figure 1:**
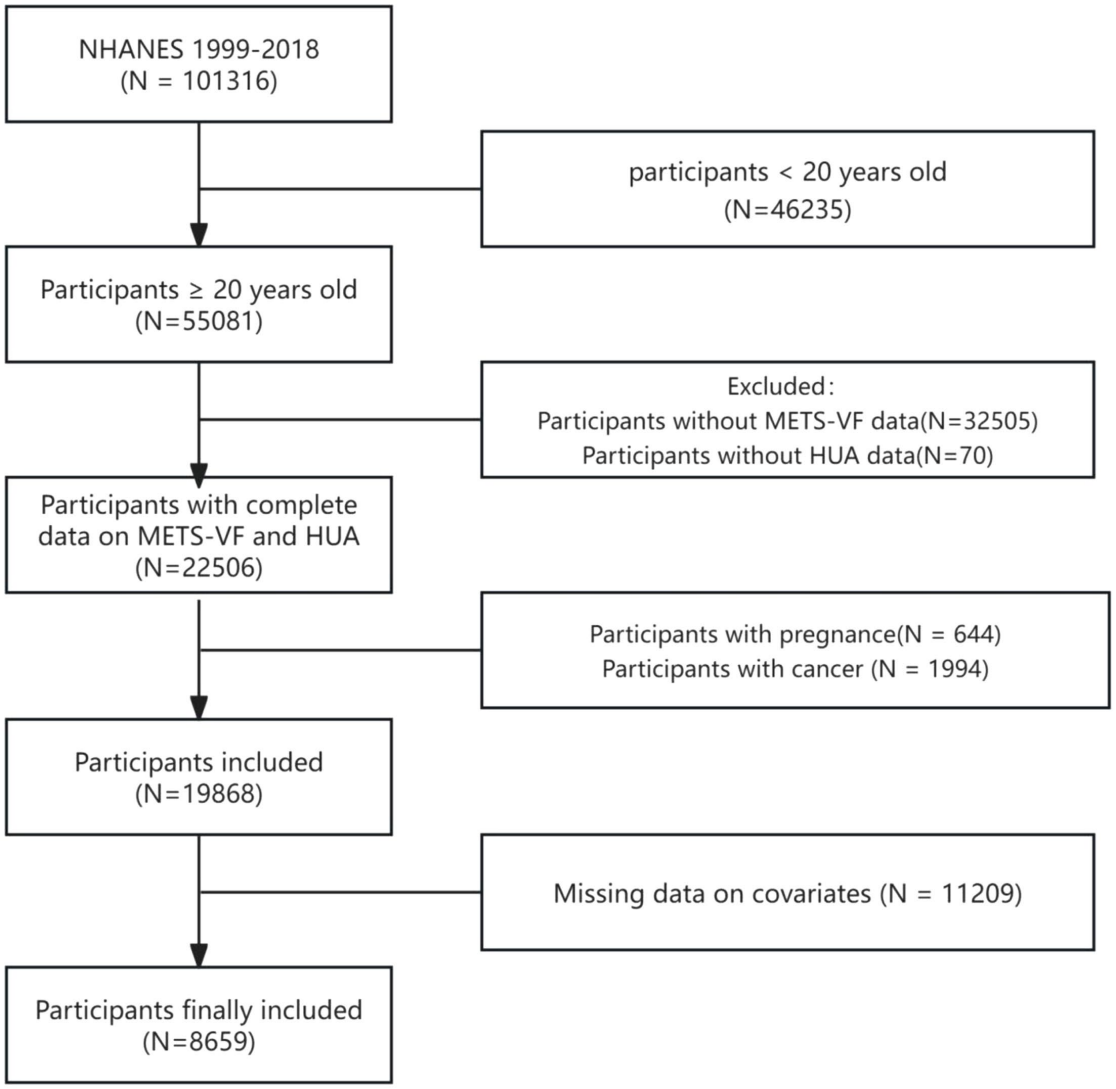
A flow chart of participants screening in NHANES 1999–2018. NHANES: National Health and Nutrition Examination Survey, HUA: hyperuricemia, METS-VF: Metabolic Score for Visceral Fat.

The National Health and Nutrition Examination Survey (NHANES) is a study to assess the health and nutritional status of a nationally representative sample of adults and children in the US. The National Ethical Review Board approved the study for Health Statistics Research, and participants provided their participation by signing an informed consent form. This study utilized a 10-cycle survey dataset from 1999 to 2018, focusing on participants with HUA. We screened participants by the following exclusion criteria: (1) individuals under the age of 20 (*n* = 46,235); (2) missing METS-VF data: BMI (*n* = 3,751), WC (*n* = 2,178), height (*n* = 0), high-density lipoprotein cholesterol (HDL-C, *n* = 2,452), triglyceride (*n* = 24,078), blood glucose (*n* = 46); (3) missing uric acid data (*n* = 70); (4) pregnancy (*n* = 644); (5) cancer diagnosis (*n* = 1,994); and (6) covariates: smoking (*n* = 18), physical activity (*n* = 9,414), dietary data (*n* = 407), blood pressure (*n* = 225), total cholesterol (*n* = 0), low-density lipoprotein cholesterol (LDL-C, *n* = 274), serology index (*n* = 16), data on urinary protein and urinary creatinine (*n* = 46), cardiovascular disease (CVD, *n* = 0), education level (*n* = 6), income-to-poverty ratio (PIR, *n* = 727), and marital status (*n* = 76). The ultimate study population consisted of 8,659 participants (Figure 1).

### METS-VF calculation

METS-VF is a new surrogate index of fat accumulation calculated from METS-IR, WHtR, BMI, age, and sex. Specialized technicians assessed the individual’s height, BMI, and WC. The Cobas 6,000 chemistry analyzer was employed to analyze HDL-C and triglyceride, while the Roche/Hitachi Cobas C311 chemistry analyzer was used to measure fasting blood glucose. The following measurements were taken: fasting blood glucose and HDL-C in mg/dl, triglyceride in mg/dl, BMI in kg/m^2^, age in years, and sex as male = 1 and female = 0. The calculation formula is as follows:


$$\mathrm{METS}-IR=\frac{\ln[(\begin{array}{l} 2\times \mathrm{Blood glucose}\left(\mathrm{mg}/\mathrm{dl}\right)\\ +\mathrm{Triglyceride}\;\left(\mathrm{mg}/\mathrm{dl}\right) \end{array}]\times BMI\left(\mathrm{kg}/m\times 2\right)}{\ln\;\left[HDL\;\left(\mathrm{mg}/\mathrm{dl}\right)\right]}$$

$$\mathrm{WHtR}=\frac{WC}{\mathrm{Height}}$$

$$\begin{array}{l} \mathrm{METS}-VF=4.466+0.011\times {\left[\ln\left(\mathrm{METS}-IR\right)\right]}^{3}+3.239\\ \times {\left[\ln\left(\mathrm{WHtR}\right)\right]}^{3}+0.319\times \mathrm{sex}\;\left(\mathrm{male}=1\mathrm{,female}=0\right)\\ +0.594\times \ln\left(\mathrm{age}\right)\; \end{array}$$

### Definition of HUA

HUA was defined as a serum uric acid threshold of 420 mmol/L or higher in men and 360 mmol/L or higher in women. We obtained serum uric acid data in the serum module with the variable name LBDSUASI.

### Covariates

According to the previous study (27), we consider the following confounding factors as covariates: (1) demographic data: age, sex, race, education level, marital status, and PIR; (2) lifestyle: smoking, alcohol consumption, physical activity, and energy intake; (3) combined diseases: hypertension, diabetes, hyperlipidemia, chronic kidney disease (CKD), and CVD; (4) serological indexes: aspartate aminotransferase (AST) and alanine aminotransferase (ALT); (5) Drug use: diuretic use. Notably, we obtained alcohol-drinking data in the dietary module. Moderate daily alcohol drinking was measured from 0–30 mg for men and from 0–15 mg for women. Physical activity was the weekly metabolic equivalent for calculation. We categorized moderate activity as the weekly metabolic equivalents of 600 to 1,200 per week.

### Statistical analysis

WTSAF4YR and WTSAF2YR were selected as the sampling weights for the weighted analysis. Continuous data were represented as weighted averages (means) and standard deviations (SDs) (i.e., mean ± SD), whereas categorical variables were expressed as weighted percentages (percentages, %). We employed chi-square and *t*-tests to compare categorical and continuous variables among distinct groups. We performed a 1:1 nearest-neighbor propensity matching score for HUA and non-HUA patients with a caliper score of 0.05. The adjusted variables were selected for age, sex, education level, marital status, smoking, alcohol consumption, diabetes, hypertension, hyperlipidemia, CKD, CVD, diuretic use, energy intake, AST, and ALT. The correlation between METS-VF and HUA before and after PMS was analyzed using three weighted logistic regression models. Model 1 does not account for any variables. Model 2 was modified to account for age, sex, and race. The Model 3 was controlled for all potential confounding variables. Sensitivity analysis included subgroup analysis, interaction test, and restricted cubic spline (RCS) analysis. The study was carried out using R Statistical Software (v4.3.1; (56)), which included the survey, rms, and MatchIt packages; *p* < 0.05 indicated statistical significance.

## Results

### Population characteristics described by the quartiles of METS-VF

The study comprised 8,659 individuals aged 20 years and older, of whom 1,465 (16.52%) were diagnosed with HUA. Among these participants, 51.21% were males. METS-VF was divided into quartiles ≤5.75, 5.75–6.26, 6.26–6.62, and > 6.62. Compared with the lowest quartile (Q1) group, individuals in the highest METS-VF group (Q4) were more inclined to be aged 40–65 years, males, Non-Hispanic White, above high school level, married, PIR ≥3.5, former smokers, those who never consumed alcohol, hypertension, diabetes, hyperlipidemia, CKD, CVD, low physical activity, more individuals using diuretics, and higher AST and ALT. There was a corresponding rise in METS-VF and HUA prevalence (Table 1).

**Table 1:** Demographic characteristics of all participants based on METS-VF quartile grouping.

Characteristic	Overall, *N* = 8,659 (100%)	Q1, *N* = 2,165 (28%)	Q2, *N* = 2,165 (26%)	Q3, *N* = 2,164 (25%)	Q4, *N* = 2,165 (21%)	*p*-value
HUA						<0.001
No	7,194 (83.48%)	2,031 (93.81%)	1,893 (87.36%)	1,724 (78.35%)	1,546 (70.41%)	
Yes	1,465 (16.52%)	134 (6.19%)	272 (12.64%)	440 (21.65%)	619 (29.59%)	
Age (years)	43.60 ± 15.51	33.19 ± 11.60	40.42 ± 13.48	46.97 ± 13.26	57.97 ± 12.57	<0.001
Age, year (%)						<0.001
20–39	3,697 (46.09%)	1,657 (75.20%)	1,178 (54.72%)	721 (34.37%)	141 (8.96%)	
40–65	3,520 (42.49%)	465 (23.54%)	812 (39.70%)	1,129 (54.55%)	1,114 (57.72%)	
≥65	1,442 (11.42%)	43 (1.27%)	175 (5.58%)	314 (11.08%)	910 (33.32%)	
Sex (%)						<0.001
Male	4,517 (51.21%)	938 (39.62%)	987 (46.17%)	1,099 (53.18%)	1,493 (71.30%)	
Female	4,142 (48.79%)	1,227 (60.38%)	1,178 (53.83%)	1,065 (46.82%)	672 (28.70%)	
Education level (%)						<0.001
Below high school	1,633 (11.39%)	274 (8.90%)	375 (11.23%)	443 (12.77%)	541 (13.36%)	
High school	1,863 (21.78%)	412 (18.50%)	470 (22.54%)	487 (22.95%)	494 (23.96%)	
Above high school	5,163 (66.83%)	1,479 (72.60%)	1,320 (66.24%)	1,234 (64.28%)	1,130 (62.68%)	
Marital status (%)						<0.001
Married/living with partner	5,294 (65.19%)	1,065 (53.53%)	1,393 (68.61%)	1,409 (70.92%)	1,427 (70.05%)	
Never married	1,796 (20.24%)	877 (37.14%)	448 (19.50%)	314 (12.29%)	157 (7.39%)	
Widowed/divorced/separated	1,569 (14.57%)	223 (9.33%)	324 (11.89%)	441 (16.79%)	581 (22.56%)	
PIR group (%)						0.046
≤1.3	2,193 (16.60%)	575 (18.62%)	542 (17.08%)	542 (16.09%)	534 (13.78%)	
1.3–3.5	3,201 (34.56%)	763 (34.59%)	805 (34.47%)	808 (34.14%)	825 (35.13%)	
≥3.5	3,265 (48.85%)	827 (46.79%)	818 (48.45%)	814 (49.77%)	806 (51.09%)	
Race (%)						<0.001
Mexican American	1,400 (6.99%)	239 (5.30%)	376 (8.43%)	398 (8.23%)	387 (6%)	
Non-Hispanic White	4,035 (71.01%)	1,049 (71.17%)	975 (68.30%)	944 (69.35%)	1,067 (76.28%)	
Non-Hispanic Black	1,713 (10.20%)	464 (11.08%)	402 (9.53%)	446 (10.72%)	401 (9.20%)	
Others	1,511 (11.79%)	413 (12.45%)	412 (13.73%)	376 (11.71%)	310 (8.52%)	
Smoking status (%)						<0.001
Never	4,879 (56.31%)	1,347 (61.21%)	1,267 (56.21%)	1,253 (58.30%)	1,012 (47.26%)	
Former	2,126 (24.81%)	286 (15.28%)	438 (21.88%)	527 (25.65%)	875 (40.74%)	
Current	1,654 (18.88%)	532 (23.51%)	460 (21.90%)	384 (16.05%)	278 (12.01%)	
Alcohol consumption (%)						<0.001
Never	5,797 (62.48%)	1,336 (57.30%)	1,422 (61.59%)	1,502 (65.22%)	1,537 (67.48%)	
Low to moderate	1,625 (20.54%)	418 (21.21%)	411 (19.86%)	400 (20.86%)	396 (20.11%)	
Heavy	1,237 (16.98%)	411 (21.50%)	332 (18.55%)	262 (13.92%)	232 (12.40%)	
Hypertension (%)						<0.001
No	5,621 (70.12%)	1,938 (91%)	1,666 (79.11%)	1,254 (61.60%)	763 (40.06%)	
Yes	3,038 (29.88%)	227 (9%)	499 (20.89%)	910 (38.40%)	1,402 (59.94%)	
Diabetes mellitus (%)						<0.001
No	7,591 (91.69%)	2,129 (98.73%)	2,085 (97.48%)	1,842 (89.60%)	1,535 (77.07%)	
Yes	1,068 (8.31%)	36 (1.27%)	80 (2.52%)	322 (10.40%)	630 (22.93%)	
Hyperlipidemia (%)						<0.001
No	2,580 (31.32%)	1,203 (54.90%)	637 (30.59%)	399 (17.91%)	341 (15.83%)	
Yes	6,079 (68.68%)	962 (45.10%)	1,528 (69.41%)	1,765 (82.09%)	1,824 (84.17%)	
CKD (%)						<0.001
No	7,568 (90.49%)	2,047 (94.99%)	1,990 (93.62%)	1,901 (90.44%)	1,630 (80.33%)	
Yes	1,091 (9.51%)	118 (5.01%)	175 (6.38%)	263 (9.56%)	535 (19.67%)	
CVD (%)						<0.001
No	8,102 (94.88%)	2,140 (99.32%)	2,096 (96.88%)	2,051 (95.31%)	1,815 (85.69%)	
Yes	557 (5.12%)	25 (0.68%)	69 (3.12%)	113 (4.69%)	350 (14.31%)	
Physical activity (%)						<0.001
Low	2,793 (31.04%)	545 (25.05%)	658 (29.48%)	745 (34.26%)	845 (37.44%)	
Moderate	1,895 (22.02%)	431 (19.94%)	442 (20.77%)	505 (23.63%)	517 (24.57%)	
High	3,971 (46.93%)	1,189 (55.01%)	1,065 (49.75%)	914 (42.11%)	803 (37.99%)	
Diuretic use (%)						<0.001
No	8,109 (95.16%)	2,143 (99.07%)	2,104 (98.08%)	2,026 (94.40%)	1,836 (86.96%)	
Yes	550 (4.84%)	22 (0.93%)	61 (1.92%)	138 (5.60%)	329 (13.04%)	
Energy, kcal/day	2,263.22 ± 985.75	2,315.19 ± 1,037.28	2,214.44 ± 990.56	2,270.55 ± 955.10	2,244.85 ± 938.97	0.049
ALT (U/L)	25.99 ± 30.65	21.11 ± 46.40	25.93 ± 23.75	28.04 ± 18.46	30.34 ± 20.17	<0.001
AST (U/L)	25.11 ± 17.92	23.20 ± 16.16	25.49 ± 25.29	25.27 ± 12.23	27.05 ± 13.94	<0.001

HUA: hyperuricemia, PIR: income-to-poverty ratio, CKD: chronic kidney disease, CVD: cardiovascular disease, AST, aspartate aminotransferase, ALT, alanine aminotransferase, METS-VF: Metabolic Score for Visceral Fat, Q: quartile.

### Characteristics of participants before and after propensity matching

**Figure 2:**
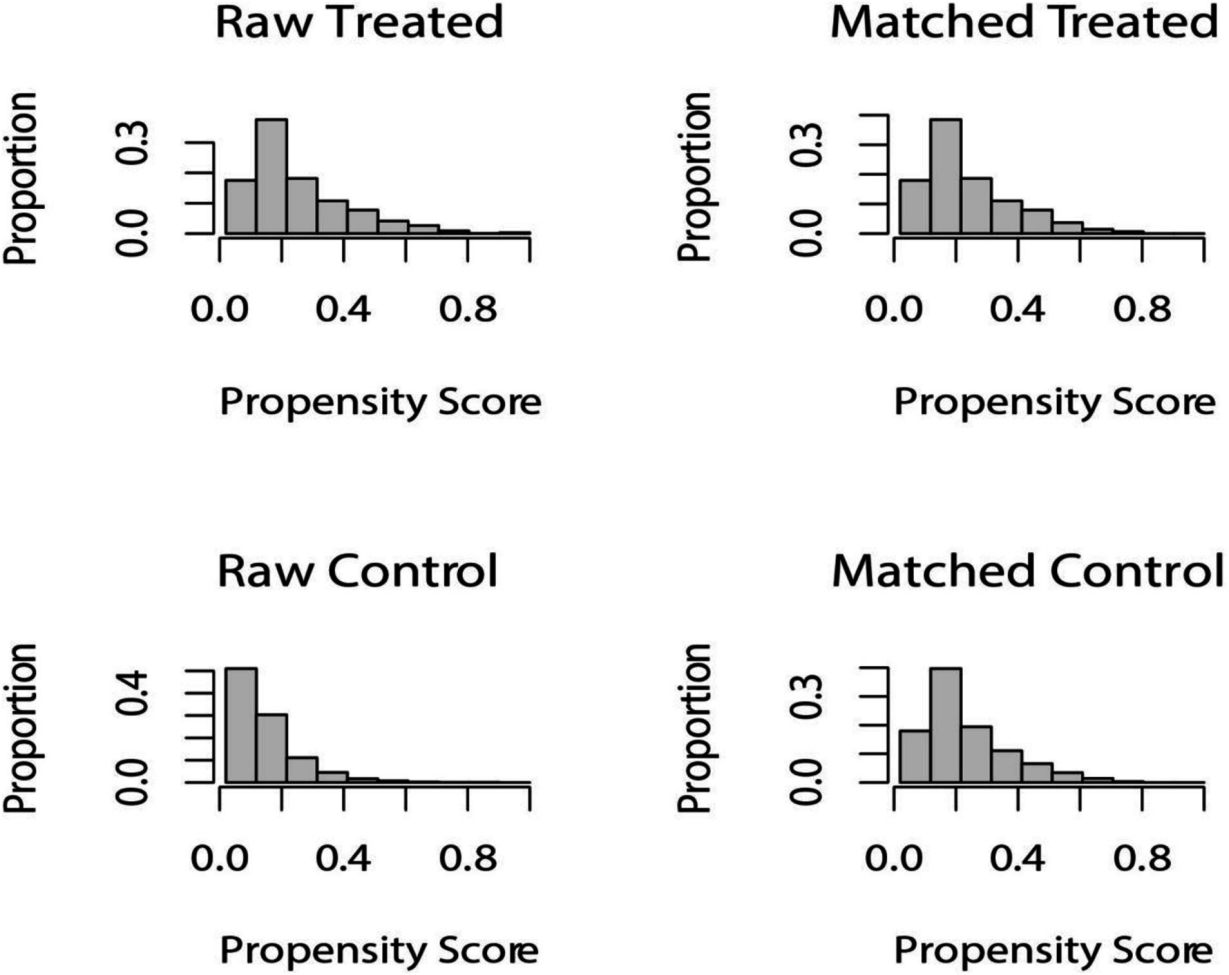
Distribution of propensity score before and after matching. Before undergoing PSM at a 1:1 ratio, raw treated units, and raw control units represented the distribution of individuals in the HUA and non-HUA groups, respectively. After matching, the two groups were represented by matched treated units and matched control units, respectively. The figure illustrates the balanced distribution of individuals in the matched groups following PSM.

After PSM 1:1 caliper matching therapy, 1,434 pairs were matched with HUA and non-HUA groups. Before PSM, the two groups were statistically different in age, sex, education level, marital status, smoking, alcohol consumption, hypertension, diabetes, hyperlipidemia, CKD, CVD, diuretic use, ALT, and AST. After PSM, no statistically significant comparisons were made between the two groups except for physical activity, energy intake, ALT, and AST. METS-VF was higher in the HUA group than in the non-HUA group, with statistical significance before and after matching (Figure 2; Table 2).

**Table 2:** Before and after PSM, characteristics of participants with and without HUA.

	Before PSM			After PSM		
Characteristic	No, *N* = 7,194 (83%)	Yes, *N* = 1,465 (17%)	*p*-value	No, *N* = 1,434 (49%)	Yes, *N* = 1,434 (51%)	*p*-value
METS-VF	5.98 ± 0.69	6.40 ± 0.49	<0.001	6.19 ± 0.63	6.39 ± 0.49	<0.001
Age (years)	43.16 ± 15.24	45.81 ± 16.64	<0.001	45.22 ± 16.71	45.63 ± 16.63	0.560
Age, year (%)			<0.001			0.60
20–39	3,173 (46.71%)	524 (42.99%)		579 (45.85%)	517 (43.42%)	
40–65	2,928 (43.04%)	592 (39.70%)		511 (37.46%)	582 (39.82%)	
≥65	1,093 (10.25%)	349 (17.31%)		344 (16.69%)	335 (16.75%)	
Sex (%)			<0.001			0.80
Male	3,612 (48.65%)	905 (64.16%)		924 (64.25%)	882 (63.73%)	
Female	3,582 (51.35%)	560 (35.84%)		510 (35.75%)	552 (36.27%)	
Education level (%)			0.025			0.847
Below high school	1,392 (11.69%)	241 (9.85%)		233 (9.31%)	239 (10.02%)	
High school	1,515 (21.24%)	348 (24.52%)		345 (24.06%)	342 (24.21%)	
Above high school	4,287 (67.07%)	876 (65.63%)		856 (66.63%)	853 (65.77%)	
Marital status (%)			0.014			0.416
Married/Living with partners	4,420 (65.81%)	874 (62.07%)		810 (60.24%)	859 (62.34%)	
Never married	1,520 (20.23%)	276 (20.26%)		316 (22.95%)	271 (20.45%)	
Widowed/divorced/separated	1,254 (13.95%)	315 (17.68%)		308 (16.81%)	304 (17.21%)	
PIR group (%)			0.185			0.442
≤1.3	1,846 (17.04%)	347 (14.36%)		358 (16.04%)	343 (14.59%)	
1.3–3.5	2,659 (34.36%)	542 (35.57%)		557 (36.94%)	529 (35.51%)	
≥3.5	2,689 (48.60%)	576 (50.07%)		519 (47.02%)	562 (49.90%)	
Race (%)			0.143			0.190
Mexican American	1,214 (7.26%)	186 (5.67%)		232 (7.67%)	184 (5.77%)	
Non-Hispanic White	3,334 (70.87%)	701 (71.74%)		690 (70.70%)	680 (71.40%)	
Non-Hispanic Black	1,373 (10.09%)	340 (10.75%)		276 (9.91%)	334 (10.81%)	
Others	1,273 (11.79%)	238 (11.83%)		236 (11.72%)	236 (12.01%)	
Smoking status (%)			<0.001			0.7
Never	4,122 (57.16%)	757 (52.04%)		760 (54.18%)	741 (52.23%)	
Former	1,692 (23.77%)	434 (30.10%)		425 (28.11%)	424 (29.69%)	
Current	1,380 (19.08%)	274 (17.86%)		249 (17.71%)	269 (18.08%)	
Alcohol consumption (%)			<0.001			0.536
Never	4,875 (63.02%)	922 (59.74%)		960 (62.33%)	904 (60.07%)	
Low to moderate	1,369 (21.07%)	256 (17.89%)		229 (16.44%)	256 (18.32%)	
Heavy	950 (15.91%)	287 (22.37%)		245 (21.23%)	274 (21.61%)	
Hypertension (%)			<0.001			0.614
No	4,945 (73.61%)	676 (52.52%)		697 (54.59%)	671 (53.30%)	
Yes	2,249 (26.39%)	789 (47.48%)		737 (45.41%)	763 (46.70%)	
Diabetes mellitus (%)			<0.001			0.461
No	6,406 (92.67%)	1,185 (86.71%)		1,183 (88.01%)	1,161 (86.92%)	
Yes	788 (7.33%)	280 (13.29%)		251 (11.99%)	273 (13.08%)	
Hyperlipidemia (%)			<0.001			0.750
No	2,315 (33.65%)	265 (19.56%)		253 (18.89%)	264 (19.59%)	
Yes	4,879 (66.35%)	1,200 (80.44%)		1,181 (81.11%)	1,170 (80.41%)	
CKD (%)			<0.001			0.660
No	6,462 (92.44%)	1,106 (80.65%)		1,093 (82.31%)	1,096 (81.60%)	
Yes	732 (7.56%)	359 (19.35%)		341 (17.69%)	338 (18.40%)	
CVD (%)			<0.001			0.975
No	6,797 (95.52%)	1,305 (91.68%)		1,284 (91.81%)	1,278 (91.85%)	
Yes	397 (4.48%)	160 (8.32%)		150 (8.19%)	156 (8.15%)	
Physical activity (%)			0.199			0.029
Low	2,293 (31.01%)	500 (31.23%)		472 (31.79%)	489 (31.19%)	
Moderate	1,555 (21.60%)	340 (24.16%)		301 (18.85%)	333 (24.20%)	
High	3,346 (47.39%)	625 (44.61%)		661 (49.36%)	612 (44.60%)	
Diuretic use (%)			<0.001			0.340
No	6,864 (96.48%)	1,245 (88.51%)		1,256 (90.24%)	1,230 (88.89%)	
Yes	330 (3.52%)	220 (11.49%)		178 (9.76%)	204 (11.11%)	
Energy, kcal/day	2,262.38 ± 985.12	2,267.43 ± 989.26	0.817	2,406.08 ± 1,101.48	2,261.27 ± 993.11	0.007
ALT (U/L)	24.24 ± 16.97	34.82 ± 64.35	<0.001	28.92 ± 25.22	33.09 ± 62.04	<0.001
AST (U/L)	24.23 ± 15.55	29.52 ± 26.42	<0.001	26.76 ± 28.24	28.20 ± 18.79	<0.001

HUA: hyperuricemia, PIR: income-to-poverty ratio, CKD: chronic kidney disease, CVD: cardiovascular disease, AST, aspartate aminotransferase; ALT, alanine aminotransferase, PSM: propensity score matching, METS-VF: Metabolic Score for Visceral Fat. Categorical variables were expressed as weighted percentages (%). Continuous variables were expressed as mean (± standard deviation).

### Correlation analysis between METS-VF and HUA

Three multivariate logistic regression models were constructed to examine the association between METS-VF and HUA (Table 3). In model 3, we controlled for age, sex, race, education level, marital status, PIR, smoking, alcohol consumption, hypertension, diabetes, hyperlipidemia, CKD, CVD, physical activity, diuretic use, energy intake, ALT, and AST. The results indicated that for every unit rise in METS-VF, the likelihood of HUA increased by 251% after accounting for all potential influencing factors [OR: 3.51 (2.88, 4.27)]. The OR for the highest level of METS-VF was 6.07 (4.39, 8.38) compared to the lowest level of METS-VF.

**Table 3:** Weighted logistic regression analysis of the connection between METS-VF and HUA.

	^a^Model 1			^b^Model 2			^c^Model 3		
Characteristic	OR	95% CI	*p*-value	OR	95% CI	*p*-value	OR	95% CI	*p*-value
Before PSM									
Continuous	3.39	2.96, 3.88	<0.001	4.34	3.64, 5.17	<0.001	3.51	2.88, 4.27	<0.001
^d^Quartile									
Q1		Ref			Ref			Ref	
Q2	2.19	1.68, 2.86	<0.001	2.48	1.91, 3.23	<0.001	2.18	1.65, 2.89	<0.001
Q3	4.19	3.26, 5.38	<0.001	5.36	4.13, 6.96	<0.001	4.32	3.24, 5.76	<0.001
Q4	6.37	5.08, 7.98	<0.001	8.82	6.68, 11.6	<0.001	6.07	4.39, 8.38	<0.001
*P* for trend		<0.001			<0.001			<0.001	
After PSM									
Continuous	1.91	1.64, 2.23	<0.001	2.48	2.05, 2.99	<0.001	2.90	2.36, 3.58	<0.01
^d^Quartile									
Q1		Ref			Ref			Ref	
Q2	1.42	1.03, 1.97	0.033	1.57	1.15, 2.16	0.005	1.72	1.24, 2.37	0.001
Q3	2.27	1.69, 3.04	<0.001	2.74	2.03, 3.71	<0.001	3.28	2.39, 4.50	<0.001
Q4	2.51	1.91, 3.29	<0.001	3.64	2.62, 5.05	<0.001	4.53	3.17, 6.46	<0.001
*P* for trend		<0.001			<0.001			<0.001	

a Model 1: adjusted for no covariates.

b Model 2: adjusted for age, gender, and race.

c Model 3: adjusted for age, gender, race, education level, marital status, PIR, smoking, alcohol consumption, hypertension, diabetes, hyperlipidemia, CKD, CVD, physical activity, diuretic use, energy intake, ALT, and AST.

d METS-VF quartiles: Q1: ≤5.75, Q2: 5.75–6.26, Q3: 6.26–6.62, Q4: >6.62.

HUA: hyperuricemia, METS-VF: Metabolic Score for Visceral Fat, OR: odds ratio, CI: confidence interval, PSM: propensity score matching, PIR: income-to-poverty ratio, CKD: chronic kidney disease, CVD: cardiovascular disease, AST, aspartate aminotransferase; ALT, alanine aminotransferase, Q: quartile.

After PSM, the connection between METS-VF and HUA remained substantial. In the fully adjusted model, the odds ratio for developing HUA was 2.90 (2.36, 3.58) for each unit of rise in METS-VF. Using Q1 as the reference, the ORs for Q2, Q3, and Q4 are 1.72 (1.24, 2.37), 3.28 (2.39, 4.50), and 4.53 (3.17, 6.46), respectively.

### RCS analysis of the METS-VF and HUA

**Figure 3:**
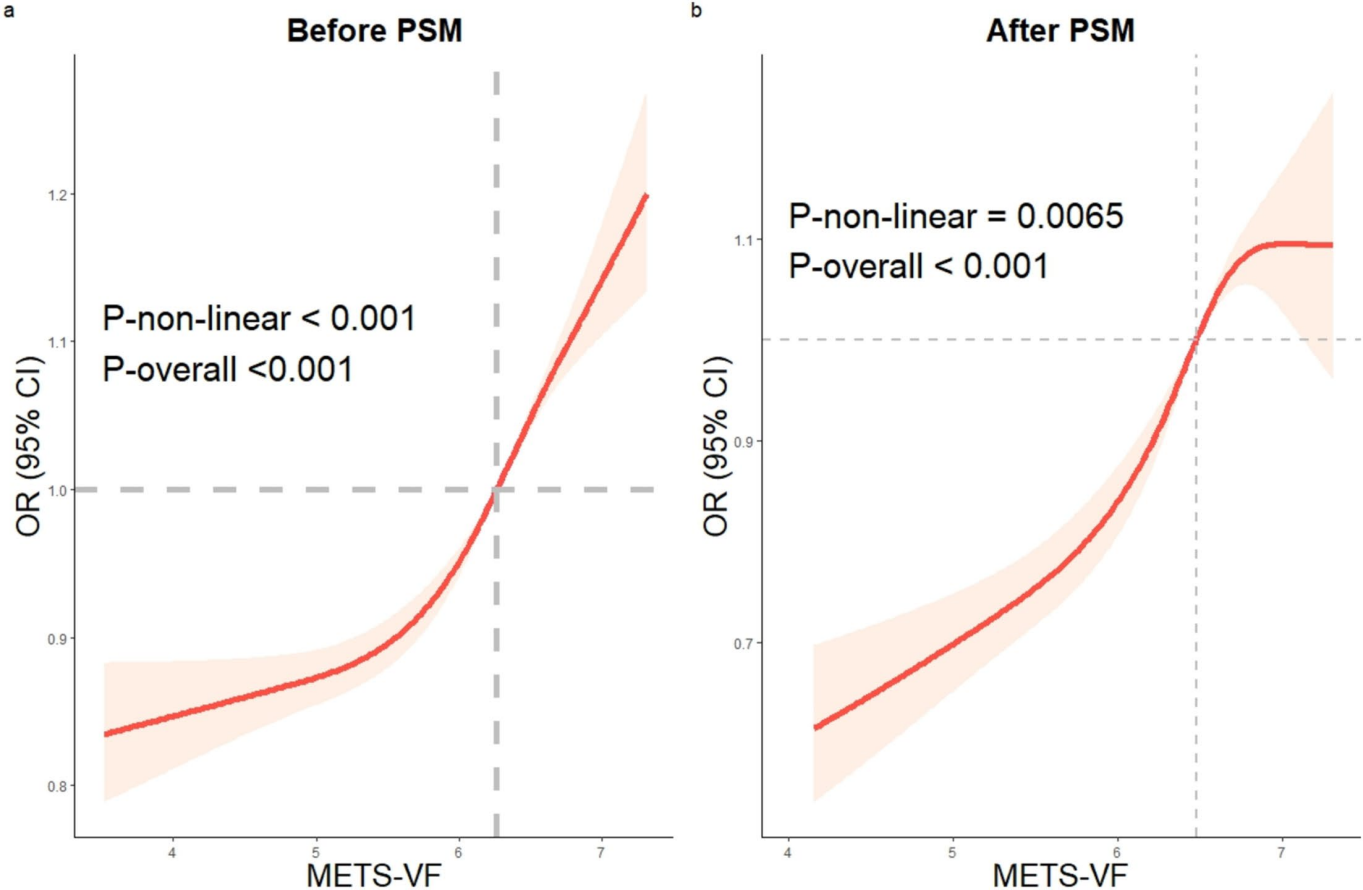
The RCS analysis of METS-VF and HUA. Before and after PSM, a non-linear correlation between METS-VF and HUA. RCS: restricted cubic spline, HUA: hyperuricemia, METS-VF: Metabolic Score for Visceral Fat, PSM: propensity score matching.

After correcting for all confounding variables, the RCS analysis of the connection between METS-VF and HUA was conducted. The study identified a non-linear positive connection between METS-VF and the incidence of HUA both before and after PSM (before PSM: *p*-non-linear <0.001; after PSM: *p*-non-linear = 0.0065) (Figure 3).

### Subgroup analysis and interaction test between METS-VF and HUA

**Figure 4:**
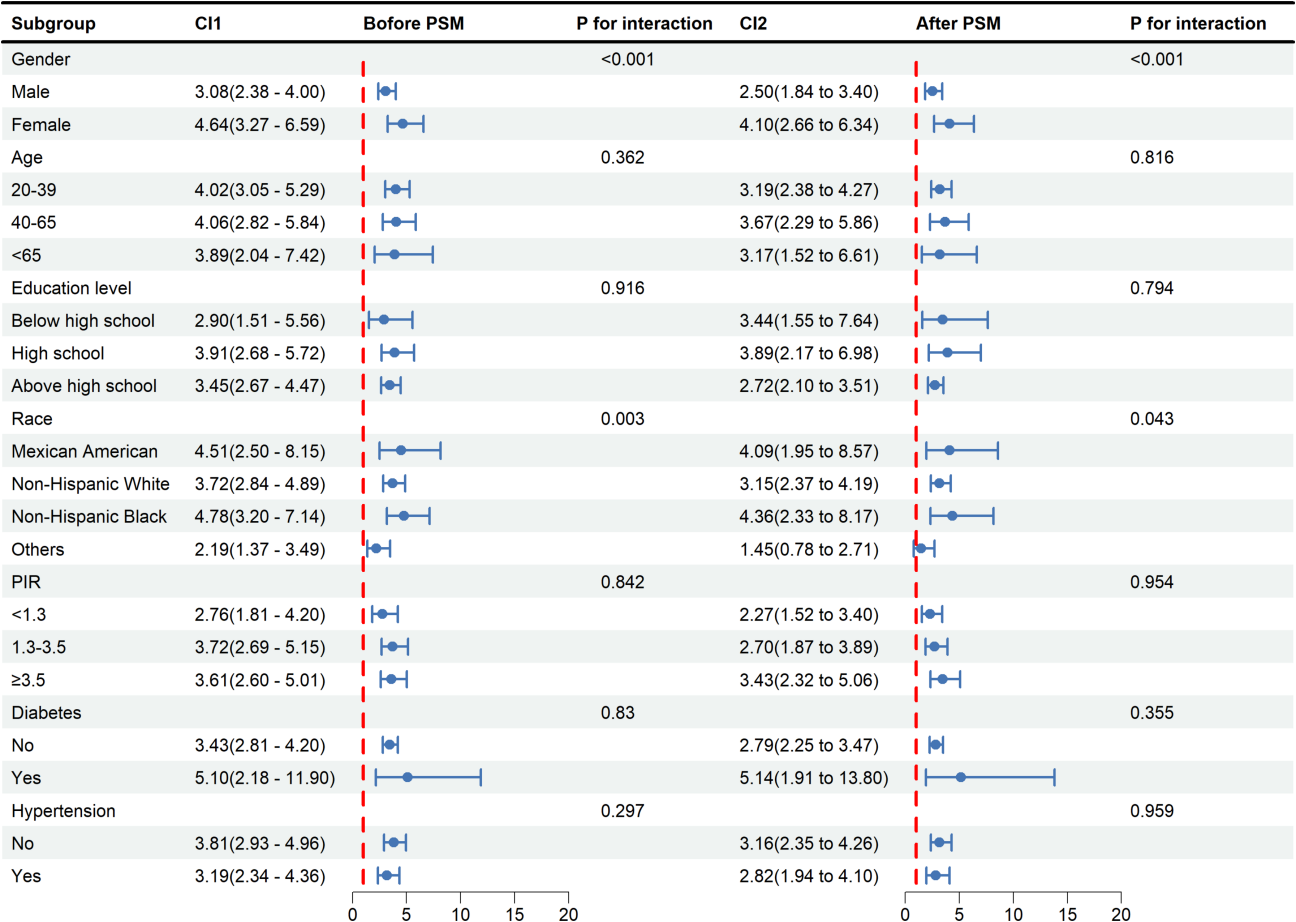
Subgroup analysis and interaction tests for the relationship between METS-VF and HUA. METS-VF: Metabolic Score for Visceral Fat, HUA: hyperuricemia, PSM: propensity score matching, CI: confidence interval, PIR: income-to-poverty ratio.

In general, consistent results before and after PSM are considered reliable (28). Before and after PSM, the relationship between METS-VF and HUA was statistically significantly different between classes, indicating that age, sex, education level, PIR, hypertension, race except for others, and diabetes all significantly affected this positive association. Interaction tests showed that the effect of METS-VF on HUA varied by sex and race (*p* < 0.05 for interaction) (Figure 4).

## Discussion

Our study was designed to examine the correlation between METS-VF and HUA. We discovered that the elevated METS-VF was substantially correlated with the increased likelihood of HUA in a cross-sectional study that involved 8,659 individuals. The results of the RCS showed a non-linear positive correlation between METS-VF and HUA. It was demonstrated that a higher likelihood of HUA was connected with elevated METS-VF in age, sex, education level, PIR, hypertension, race except for others, and diabetes subgroups. Interaction tests revealed that the effect of METS-VF on HUA varied by sex and ethnicity. The consistent results before and after PSM underscored the robustness of our findings.

Obesity as a recognized risk factor for HUA has prompted researchers to focus on effective prevention strategies for HUA in the obese population (29). Research has demonstrated that HUA is linked to adipose distribution (30). Consequently, it is imperative to conduct a precise evaluation of the actual obesity status of individuals. An elevated BMI is linked to an elevated incidence of HUA (31). Palmer et al. discovered that an elevated BMI contributes to the development of uric acid levels and HUA (32). In a Japanese cross-sectional study, Tanaka et al. discovered that BMI remained highly correlated with uric acid even after taking into account genetic and family environmental factors (33). Yamashita et al. established that weight loss can lead to an improvement in levels of uric acid in obese people (34). Unfortunately, BMI cannot distinguish between lean and adipose tissue mass (35). Accumulating visceral fat has a more harmful impact on uric acid metabolism compared to BMI and subcutaneous fat accumulation (36). Although both WC and WHtR are predictive of HUA (37), visceral obesity is susceptible to race, sex, and age (35, 38). Furthermore, high insulin levels caused by IR might limit uric acid excretion while increasing uric acid production, resulting in uric acid accumulation (39, 40). METS-VF combines age, sex, WhtR, and METS-IR variables and is expected to serve as a response indicator for HUA. In the Chinese population, a longitudinal association study demonstrated that a higher METS-VF increased the likelihood of HUA (41). Our study confirmed a positive correlation between METS-VF and HUA in the US population.

Considering that the reduction of visceral fat can mitigate the risk of HUA, we propose that public health initiatives should concentrate on lifestyle modifications to decrease visceral fat, potentially preventing HUA and enhancing overall health. Takeshita et al. discovered in a murine model that prolonged voluntary exercise reduced visceral fat in mice (42). Excessive fructose consumption can elevate intracellular cortisol levels, resulting in enhanced fatty acid release from subcutaneous adipocytes, hence facilitating more substrate storage in visceral adipose tissue (43). De Amicis et al. demonstrated that evening-type circadian rhythms may be a significant factor in abdominal obesity and its visceral component (44). Furthermore, a population study conducted by Nakanishi et al. revealed that women who smoked exhibited a higher propensity for visceral fat accumulation (45). Consequently, we underscore that alongside identifying individuals at elevated risk for visceral obesity to avert hyperuricemia, it is imperative to prioritize establishing a healthy lifestyle and sound dietary practices.

Visceral fat accumulation causes an inflow of plasma-free fatty acids into the liver and hepatic portal vein, which boosts triglyceride synthesis and, through altering purine metabolism, causes an increase in the generation of uric acid (46, 47). Matsuura et al. discovered that in obese males, visceral fat increase had a more substantial impact on uric acid metabolism (48). Also, uric acid produces oxidized lipids and inflammatory mediators in adipocytes by activating NADPH oxidase, which, in turn, induces and exacerbates IR (39). IR leads to a significant accumulation of insulin, which can reduce renal excretion of uric acid (39).

The results of this study indicate that sex and racial disparities have significant significance for the relationships examined. The METS-VF formula includes age and IR, which contribute to the accumulation of visceral fat. As age advances, the distribution of fat shifts from being located just beneath the skin to being stored around internal organs, and visceral fat accumulation increases at a rapid pace (49, 50). Studies have shown a gradual increase and centralization of fat mass with age in women (51). Visceral adipose tissue area is significantly increased in postmenopausal women compared to premenopausal women (52). Additionally, in a population-based study, researchers discovered that both abdominal subcutaneous fat and visceral fat were associated with IR. They confirmed that the accumulation of visceral fat is particularly harmful in women (53). These factors may explain the stronger correlation between METS-VF and HUA risk in women. Differences in body fat distribution in different racial groups may be related to biological differences, sociocultural, and socioeconomic status, health behaviors, and neighborhood environments (54, 55). In conclusion, racial differences should be considered when taking measures to prevent HUA caused by visceral fat obesity.

### Study strengths and limitations

The connection between METS-VF and the probability of HUA development in the US population has been thoroughly investigated in this study. This study gives vital information about the potential link between these two disorders and the diagnosis and treatment of HUA. Nevertheless, our study has drawbacks. First, this study could not prove a causal link between METS-VF and HUA because of its cross-sectional methodology. Second, self-reported food and lifestyle data in the NHANES database include limitations, such as memory and social desirability bias. These biases may impact the accuracy and reliability of the data and should be taken into account when evaluating the study’s conclusions. Third, studies have indicated that consuming fructose and purine-rich foods can result in hyperuricemia. The study could not rule out the possibility that dietary and other factors could interfere with the results. Fourth, the NHANES data are representative of the US population. The study’s findings may not apply to other populations due to lifestyle, diet, and genetics differences. Finally, the METS-VF assessment was not validated by objective laboratory measurements; therefore, more research is needed to understand this correlation.

## Conclusion

Our research demonstrated a consistent and robust link between METS-VF and the incidence of HUA. Furthermore, our study has the potential to offer novel insights into the prevention and treatment of HUA.

## Data Availability

The original contributions presented in the study are included in the article/supplementary material, further inquiries can be directed to the corresponding author.

## Glossary

HUA: hyperuricemia
METS-VF: Metabolic Score for Visceral Fat
NHANES: National Health and Nutrition Examination Survey
PSM: propensity score matching
RCS: restricted cubic spline
US: United States
BMI: body mass index
WC: waist circumference
WHtR: waist-to-height ratio
METS-IR: Metabolic Score for Insulin Resistance
HDL-C: high-density lipoprotein cholesterol
PIR: income-to-poverty ratio
CVD: cardiovascular disease
CKD: chronic kidney disease
AST: aspartate aminotransferase
ALT: alanine aminotransferase
LDL-C: low-density lipoprotein cholesterol
OR: odds ratio
TyG: triglyceride-to-glucose
CI: confidence interval
Q: quartile
IR: insulin resistance
